# Foundations of cumulative culture in apes: improved foraging efficiency through relinquishing and combining witnessed behaviours in chimpanzees (*Pan troglodytes*)

**DOI:** 10.1038/srep35953

**Published:** 2016-10-24

**Authors:** Sarah J. Davis, Gillian L. Vale, Steven J. Schapiro, Susan P. Lambeth, Andrew Whiten

**Affiliations:** 1 Centre for Social learning and Cognitive Evolution, School of Psychology & Neuroscience, University of St Andrews, St Andrews, KY16 9JP, Scotland; 2 National Center for Chimpanzee Care, Department of Veterinary Sciences, Michale E. Keeling Center for Comparative Medicine and Research, the University of Texas MD Anderson Cancer Center, Bastrop, TX 78602, USA

## Abstract

A vital prerequisite for cumulative culture, a phenomenon often asserted to be unique to humans, is the ability to modify behaviour and flexibly switch to more productive or efficient alternatives. Here, we first established an inefficient solution to a foraging task in five captive chimpanzee groups (*N* = 19). Three groups subsequently witnessed a conspecific using an alternative, more efficient, solution. When participants could successfully forage with their established behaviours, most individuals did not switch to this more efficient technique; however, when their foraging method became substantially less efficient, nine chimpanzees with socially-acquired information (four of whom witnessed additional human demonstrations) relinquished their old behaviour in favour of the more efficient one. Only a single chimpanzee in control groups, who had not witnessed a knowledgeable model, discovered this. Individuals who switched were later able to combine components of their two learned techniques to produce a more efficient solution than their extensively used, original foraging method. These results suggest that, although chimpanzees show a considerable degree of conservatism, they also have an ability to combine independent behaviours to produce efficient compound action sequences; one of the foundational abilities (or candidate mechanisms) for human cumulative culture.

Culture has been defined as “group-typical behaviour patterns shared by members of a community that rely on socially learned and transmitted information” (p.151^1^). The ability to build upon or ratchet up on such cultural behaviours, creating cumulative cultural change^2^, can lead to substantial gains in productivity or efficiency, well exemplified in its elaboration in humans^3^. Whilst the ability to socially learn behaviours (defined as “learning that is influenced by observation of, or interaction with another animal (typically a conspecific) or its products” (p.207^4^) may be common across many animal taxa^5^,^6^,^7^,^8^,^9^, cumulative culture is limited or, according to some authors, absent in non-human animals^2^,^3^,^10^. This is most striking when we compare our human cultures with those of arguably the second most cultural species, our closest living relative, the chimpanzee (*Pan troglodytes*)^11^.

Chimpanzees exhibit the greatest number of traditions outside of the human species, across foraging, tool use and social behaviours, with each chimpanzee group distinguished by their own particular cultural profile^11^. Yet, there is little evidence for cultural accumulation on these traditions (see ref. 12). Various factors may contribute to the stasis of chimpanzee culture, such as relevant socio-cognitive adaptations^13^, low fidelity social learning mechanisms^14^, or failure to employ appropriate learning heuristics^15^,^16^. However, cumulative culture ultimately requires the ability to change established behaviours in order to adopt more efficient or productive ones; that is, in order to upgrade solutions, an individual must possess the behavioural flexibility to relinquish, modify and build on prior solutions. Behavioural inflexibility may therefore, in and of itself, limit the evolution of culture. With behavioural flexibility defined as “the continued interest in and acquisition of new solutions to a task, through either innovation or social learning, after already having mastered a previous solution” (p.447^17^), a lack of such flexibility has been found in several experiments with chimpanzees. Marshall-Pescini and Whiten^16^ found that young chimpanzees failed to cumulatively modify their foraging efforts by building on their exisiting behaviours despite witnessing a more productive solution. Yet, the more complex behaviour could be acquired if participants had no prior knowledge of the less lucrative foraging technique. This led the authors to suggest that chimpanzees are behaviourally conservative, since reported in several further studies^13^,^18^,^19^,^20^,^21^ (see also ref. 22); in simple terms, chimpanzees tend to become ‘stuck’ on known behaviours despite availability of superior alternatives.

These results appear inconsistent with other findings such as that of Horner and Whiten^23^, where chimpanzees ‘streamlined’ their behaviours after witnessing inefficient options used by others. However, this involved omitting elements^24^,^25^, as opposed to the additive, ratchet effect required for cumulative culure^2^. Similarly, following social demonstrations in a juice acquiring task, Yamamoto, Humle and Tanaka^26^ found that chimpanzees switched from using a straw as a dipping tool to exploiting a more efficient sucking function, but this also did not involve additive ratcheting. Such findings are in line with records of behavioural modification in the wild^27^,^28^,^29^,^30^,^31^ (see also ref. 32), as well as more recent experiments demonstrating payoff-related variation in simple behaviour, such as depositing ‘tokens’ in novel locations to increase food reward value^33^,^34^.

From studies examining behavioural change in humans, we might expect at least two factors to have differential effects on behavioural flexibility: the extent to which behaviour has been practiced, and the complexity of the behaviour involved^35^,^36^,^37^. As cultural traditions are often well-established and long-held behaviours, and are also sufficiently complex to necessitate social learning to acquire them, it may be important to consider how well-ingrained the behaviour to be modified is when extrapolating results to chimpanzees’ potential for cumulative culture. Evidence now exists that chimpanzees can recognise and adopt superior variants of behaviours which are simple and conceptually similar to existing routines^33^,^34^. Chimpanzees can also relinquish old solutions and build on very simple behaviours to form action sequences when these sequences are within most chimpanzees’ repertoires^38^, as well as relinquish behaviours that have been performed but not yet adopted as a reliable foraging strategy^23^,^26^. However, the extent to which chimpanzees can modify, relinquish or build-upon well-established, cognitively more complex behaviours, those that perhaps mirror cultural behaviours more closely, remains to be established^13^,^16^.

In the present studies, we investigated chimpanzees’ ability to build upon socially acquired, complex behaviour in the context of improving efficiency. Of particular interest is whether a chimpanzee can benefit by witnessing a more efficient behaviour used by a conspecific compared to one they currently reliably employ to achieve the same goal, and flexibly switch to using this more efficient behaviour.

A transparent puzzle box (Fig. 1) was used (hereafter ‘Serialbox’) from which a valued token could be extracted (later exchanged for a food reward) via either of two alternative operations differing in efficiency, with the inefficient method (Supplementary Video 1) more labour intensive and taking longer to complete. The efficient method (Supplementary Video 2) involved partial use of behaviours common to the inefficient method, along with the addition of a novel behaviour. The efficient method therefore involved not only streamlining the inefficient method by a *subtractive* process (noted in some studies of cumulative culture)^24^,^25^, but also the *addition* of a novel behavioural element to an established sequence, that is, a ratcheting up on behaviour^2^. Participants across five groups were initially trained to extract a valued token from the transparent Serialbox via a multi-stepped, repetitive, inefficient process (Fig. 1). To strengthen ecological validity when assessing chimpanzees’ cumulative cultural capabilities, this extraction process was completed a minimum of 20 times over several sessions until it became a reliable and ingrained response. Three groups (‘social information’ groups) subsequently witnessed a conspecific model using the more efficient solution described in Fig. 1 and more fully in Methods below. Following repeated social demonstrations, the behaviour of participants was examined over ten hours of open diffusion, monitoring any spread of the more efficient technique, to better simulate the diffusion of behaviours in a culturally relevant context^39^.

We hypothesised that if chimpanzees could recognise a solution more efficient than the one they were currently employing and were able to switch to this, they should do so once they witnessed the actions of the model, regarded as a simulated ‘innovator’^40^. To assess how readily chimpanzees could themselves innovate and switch to the efficient method without the need for social information, we trained two control groups to use the inefficient method but did not expose them to the efficient method through a trained conspecific (‘non-seeded’ groups). To investigate how naïve chimpanzees might solve this extractive problem when they did not have an established solution to the puzzle, the Serialbox was introduced to one additional control group who were not initially trained to extract via the inefficient method (‘naïve’ group). For this group, the problem could be solved by using either the efficient or inefficient strategy.

## Experiment 1: Results

Due to limited sample sizes, data were analysed using non-parametric methods with exact P values reported. Effect sizes were calculated using the *Z* score of the test statistic such that *r* = *Z*/√*N*, where *N* was the total number of observations included in the analysis. An analysis of interrater reliability using Cohen’s kappa found excellent agreement (κ = 1) between two coders’ judgement of whether the participant was extracting via the inefficient or the efficient method.

### Participant inclusion and extractions across training and test phase

Eleven individuals in the ‘social information’ groups and eight in ‘non-seeded’ control groups met criterion for inclusion in the study (a minimum of 20 inefficient extractions; see Table 1 for participant demographics; Supplementary Table S1 for behaviours in the training and test periods; Supplementary Table S2 for relative efficiency of the two extraction techniques). There was no difference in the acquisition of the inefficient method between the ‘social information’ and ‘non-seeded’ individuals in terms of number of extractions made during the training period (Mann Whitney *U* = 36, P = 0.529; Supplementary Table S1).

Within the ‘social information’ groups, to analyse any growing behavioural proficiency, the mean time taken across the first ten extractions using the inefficient method was compared to the mean time taken across the last ten inefficient extractions, using a one-tailed Wilcoxon signed rank test. If an individual did not extract 20 times during the testing period, the mean times taken for inefficient extractions either side of the median extraction were calculated and compared. Individuals became significantly more proficient at the inefficient method over this test period (*Z* = −2.803, *n* = 10, P = 0.001, *r* = −0.63), with a median reduction in extraction latency from 47.5 to 26.2 seconds.

### Switching behaviours

Across this testing period (‘E1’), nine of the 11 individuals in the ‘social information’ groups and all individuals in the ‘non-seeded’ groups continued to exclusively use the inefficient method established during the training period (‘E0’) to extract the token.

To test for switching behaviour at the individual level, following van Leeuwen *et al*.^34^, the number of inefficient and efficient extractions performed during E0 and E1 were compared using a one-tailed Fisher’s exact test. Two individuals (from separate groups) demonstrated a significant change of behaviour within this period, switching to using the efficient solution (Individual *Se*: E0_0,21_, E1_10,16_, P = 0.001; Individual *Sa*: E0_0,22_, E1_179,0_, P < 0.0001: subscripts represent frequencies of efficient and inefficient methods respectively).

### ‘Naïve’ group

One individual, *Jy,* discovered and used the efficient method within two hours of interaction with the Serialbox. Individual *Ua* observed *Jy*’s efficient method five times; following three initial failed attempts to open the door, she successfully used the efficient method to extract the token in a subsequent test session. Before *Ua* witnessed use of the efficient method, she had unsuccessfully interacted with the apparatus, exploring only the holes and lids. Two other individuals witnessed the use of the efficient method just one and five times each and never successfully extracted the token. There was no discovery of the elaborate, inefficient method.

## Experiment 1: Discussion

When chimpanzees used a well-established but laborious solution to successfully gain rewards, most were not seen to further explore alternatives, or to capitalise on social information available about a more efficient approach. The central finding from Experiment 1 was thus of a remarkable degree of conservatism, expressed in perseverance with a well-rehearsed routine despite witnessing a more efficient alternative modelled by another chimpanzee. Such conservatism has been documented in a series of other recent chimpanzee studies^13^,^16^,^18^,^19^,^20^,^21^. By contrast, in the ‘naïve’ group, the efficient method was discovered, if by only a single persistent individual, and was later adopted by another chimpanzee. The results thus tentatively suggest that having a prior solution may in itself hinder adoption of a superior alternative^16^,^18^. Such conservatism may have some adaptive value insofar as switching to an alternative may be costly, either through cognitive demands inherent to learning or potential loss of reward through lack of expertise in this method^41^,^42^. In fact, chimpanzees, who at the start of the testing period were already well practiced at the inefficient method, effectively halved the time taken to successfully extract the token across the testing period. This indicates growing expertise and skill proficiency in their behaviour, and supports previous findings that skill mastery may hinder behavioural change^16^,^18^.

To further investigate the limits of behavioural conservatism, in Experiment 2 the disparity in efficiency of behaviours was increased such that the inefficient method became not only an unreliable means of foraging but even when successfully employed, the latency to extraction from point A was typically far higher than for B. In addition, the alternative behaviour needed for extraction at point B was reduced to a single element and did not require use of parts of the inefficient method, so subjects had only to relinquish an established solution and adopt a novel one-stepped alternative with no ratcheting on prior behaviours.

## Experiment 2: Relinquishing a highly inefficient solution

**Figure 1:**
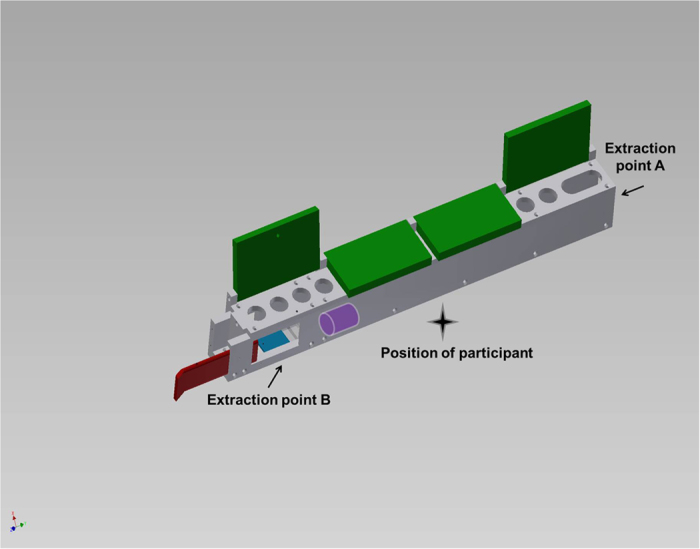
Serialbox. Along the length of the transparent Serialbox were four compartments. Each compartment had a hinged lid on top which could be lifted open (coloured here in green for image clarity; in reality all parts were transparent). Under each lid were four finger holes that permitted an object (depicted as a purple cylinder) initially provisioned in the left-most compartment to be pushed the length of the apparatus. This object could then be extracted through an opening at the other end (‘Extraction point A’). This was the inefficient method in Experiments 1 and 3. A small door spanning two thirds of the first compartment (coloured here in red for clarity) was fitted on the chimpanzee side of the apparatus and could be pulled open using a handle protruding from the outside of the box to give alternative and quicker access to the left-most compartment (‘Extraction point B’), where the token was initially positioned. This, in combination with lifting the lid of the left-most compartment and using the underlying holes to manoeuvre the token to extraction point B, was the efficient method in Experiments 1 and 3. The blue square shown in the left-most compartment depicts the indent in the floor in which the token was placed throughout Experiment 2.

The movement of the token along the length of the apparatus to extraction point A was impeded by placing the token in an indentation in the floor, directly behind extraction point B (Fig. 1), so movement of the token towards A was more awkward to initiate. However, the token could now be extracted from point B solely by just pulling the door open. Raising lids and using finger holes was unnecessary. Accordingly, this experimental manipulation made the inefficient method more so, and the efficient method yet easier, enhancing the contrast between them (Supplementary Table S2).

The 19 subjects who had met criterion for inclusion in the ‘social information’ and ‘non-seeded’ groups were all given a further ten hours of opportunity for solution and open diffusion with the inefficient method partially blocked in this way. Following Yamamoto *et al*.^26^, if individuals in the ‘social information’ groups failed to switch, they were provided with salient human demonstrations of the efficient method by SJD after this second period of open diffusion, because our question is not about chimpanzees offering such models, but rather how chimpanzees respond to such models when available. The ‘naïve’ group was not included in Experiment 2 as not only were they already exclusively using the efficient method of extraction, but their initial inclusion was designed primarily to investigate how solution naïve chimpanzees would approach this problem.

## Experiment 2: Results

### Extractions within the test period

In the ‘social information’ groups, the chimpanzee models demonstrated a 100% success rate of token extraction via the efficient method; in contrast, use of the inefficient method had a median success rate of only 25% (range 0–93%) (Supplementary Tables S1 and S2: a failed attempt was one in which a participant manipulated the Serialbox but subsequently left the apparatus without successfully extracting the token). Success rate became significantly lower in Experiment 2 (E2) compared to Experiment 1 when using the inefficient method (One-tailed Wilcoxon Signed ranks test *Z* = −2.84, *n* = 10, P = 0.001, median_E1_ = 100%, median_E2_ = 25%, *r* = −0.64). If participants were successful in extracting the token via the inefficient method, latency to extraction was almost two and a half times longer than a successful extraction in Experiment 1 (E1 median = 33.6 seconds, range = 24.5–51.8; E2 median = 83 seconds, range 66.1–556; See Supplementary Table S2 for comparisons with models’ efficiency).

In the ‘non-seeded’ groups, one individual now discovered and used the easier efficient method (Individual *Kt*), and was witnessed by two other individuals, *Na* and *Ae*. These two did not then acquire the method; however, they had observed *Kt* only three and two times respectively. No other individual was observed to use the efficient method in the ‘non-seeded’ groups, with success rate dropping for all other participants (median success rate of 14.3%, range 0–50%). Success rate was significantly lower in E2 than in E1 for those using the inefficient method in the ‘non-seeded’ groups (One-tailed Wilcoxon Signed ranks test *Z* = −2.38, *n* = 7, P = 0.008, median_E1_ = 100%, median_E2_ = 14.3%, *r* = −0.64). Success rate for those using the inefficient method did not differ between the ‘social information’ and ‘non-seeded’ groups (Mann Whitney *U* = 28, *n* = 17, P = 0.494).

### Switching behaviours

**Figure 2:**
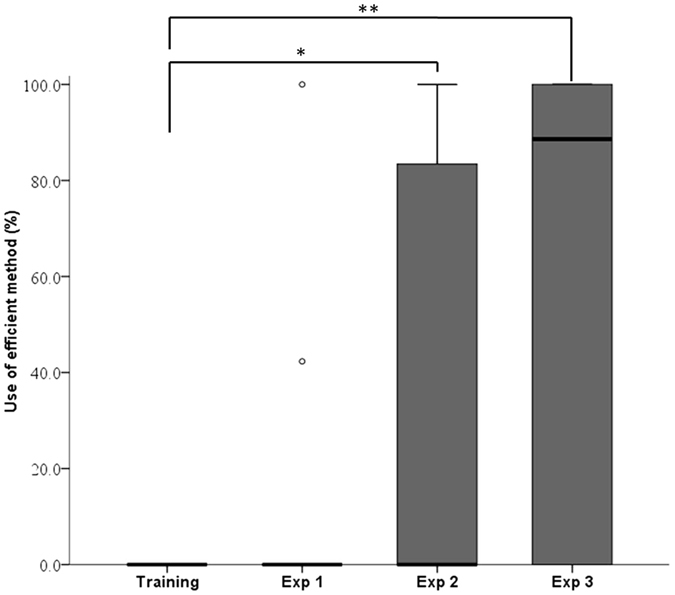
Percentage use of efficient method in Training and Experiments 1, 2 and 3 for individuals in the ‘social information’ groups. The line represents the median, the bottom and top of each box indicate the 25^th^ and 75^th^ percentile respectively, the whiskers show the minimum and the maximum values that are not considered outliers (i.e. values > 1.5 times the interquartile range from the 25^th^ or 75^th^ percentile), outliers are represented by circles with values over three times the 75^th^ percentile value. *Indicates a P value of less than 0.05 and **less than 0.01.

To assess switching behaviours in the ‘social information’ groups, the percentage of efficient extractions [efficient extractions/(efficient extractions + inefficient extractions) × 100] observed throughout E2 for each participant was compared with the percentage of efficient extractions observed during E0, using a one-tailed Wilcoxon signed rank test. There was now a significant switch, with five individuals in the ‘social information’ groups switching from the inefficient method to using the more efficient method that continued to be demonstrated by the model [*Z* = −2.023, *n* = 11, P = 0.031, median_E0_ = 0% (mean = 0%), median_E2_ = 0% (mean = 36.1%), *r* = −0.43; Fig. 2].

### Human demonstrations

After additional human demonstrations (median demonstrations given = 12, range = 10–17), four additional participants from the remaining six switched to using the efficient method in the ‘social information’ groups.

### Use of efficient method in ‘social information’ and ‘non-seeded’ groups

**Figure 3:**
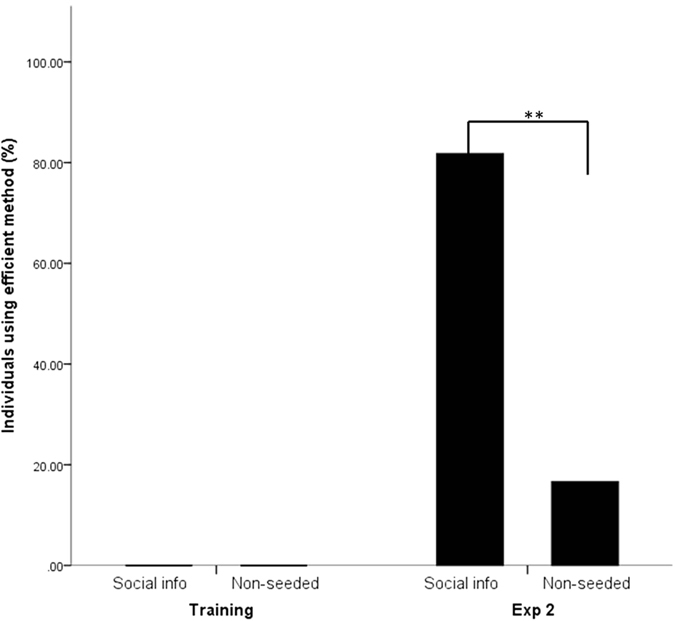
Percentage of individuals within the ‘social information’ groups and ‘non-seeded’ groups who used the efficient method across Training and Experiment 2. **Indicates a P value of less than 0.01.

To determine the role of social information in behavioural upgrading, a one-tailed Fisher’s exact test (applied due to expected values less than 5) compared the frequency of chimpanzees using the alternative method between those in ‘non-seeded’ groups and the ‘social information’ groups. A significant association was found between exposure to sustained social information and whether or not individuals switched to using the efficient alternative (P = 0.005) (Fig. 3). Based on the odds ratio, the odds of switching were 31.5 times higher for those in the ‘social information’ groups than those in the ‘non-seeded’ groups. As noted above, the two individuals who observed *Kt* in the ‘non-seeded’ group performing the efficient method did not acquire it, but they observed only three and two times respectively, whereas those in the ‘social-information’ groups had a median of 31 observations before acquisition (range 15–169; Supplementary Table S3).

## Experiment 2: Discussion

In all, nine of the 11 chimpanzees in the ‘social information’ groups were eventually able to flexibly change their behaviours by relinquishing their mastered technique and switching to a novel one. We infer that this was due to the greater contrast between participants’ inefficient use of extraction at point A and the more efficient use of extraction at point B displayed by the model, a contrast that involved differences in both latency to extraction and proportion of successful extractions.

An alternative possibility, that the changes occurred because of the more extended time frame of adding E2 to E1, affording more observations of the model, can be rejected for several reasons. First, E1 involved a long period in which any switching at all was rare, and moreover, participants not switching in E1 persevered with their inefficient technique despite both multiple observations of the model (median 18 observations, range 11–46) and multiple token extractions using their inefficient method (median 18 attempts, range 4–119 for those that switched in Experiment 2). In addition, among chimpanzees who did switch at some point, the number of observations of the efficient method did not predict the number of manipulations they would take before switching (final two columns in Supplementary Table S3). Given these considerations and that (i) only two participants were seen to open the door at point B in E1, and critically, (ii) no other individual was observed to make any persistent attempts to open the door until their behaviours became highly inefficient in E2, we conclude that the switch in behavioural strategy in E2 can be ascribed to the change in the relative efficiency of the options that were experimentally engineered between E1 and E2.

Five of the switching chimpanzees showed relatively low levels of behavioural conservatism, with two having previously upgraded their behaviours in E1, the other three adopting the alternative once their own approach became highly inefficient in E2. This was clearly facilitated by social information, as demonstrated by a lack of switching (bar one individual) in the ‘non-seeded’ groups. The social learning involved may have relied on only relatively simple processes such as stimulus enhancement (of token extraction at point B), or more complex ones, like emulation or imitation, and our study was not designed to discriminate among these. In any case, stimulus enhancement or any other social learning was insufficient for change despite extensive exposure in Experiment 1; it had effects only when the contrast in efficiency became more extreme.

Other chimpanzees still displayed a high degree of behavioural conservatism, in line with previous research^13^,^16^,^18^,^19^,^20^,^21^, showing a difficulty in inhibiting use of a highly inefficient established behaviour, with varying levels of perseveration. This was most evident in the ‘social information’ groups, where despite many observations of a far more efficient alternative, six individuals continued in their old behaviour for some time, with four only switching behaviours following salient social information engineered though human demonstrations, and the two remaining individuals never relinquishing their inefficient solutions.

There was also very little exploratory behaviour in the ‘non-seeded’ groups, with only one individual discovering the efficient method. Despite witnessing the efficient solution, two individuals within the ‘non-seeded’ groups never attempted this alternative method. This was most likely due to their more limited and inconsistent exposure to demonstrations of this method, and highlights again the conservative nature of chimpanzee behaviour. Although there was no direct relationship between the number of observations of the model and number of manipulations taken before switching, no individual within the ‘social-information’ groups was seen to switch after as few demonstrations as experienced by these ‘non-seeded’ individuals, indicating the potential need for relatively sustained social information across repeated attempts to solve the Serialbox. This mirrors findings in humans whereby trial and error learning interacts with repeated exposure to socially available alternatives to produce behavioural change^43^.

Whilst these results show some degree of behavioural flexibility, it remained to be seen whether chimpanzees could express such flexibility in a cumulative fashion; that is, could chimpanzees “add an existing technique used in a different context, or an entirely novel technique, to an existing technique, and integrate them functionally” (p. 181^44^): could they now integrate the efficient method they had acquired (door pull and extraction at point B) with behavioural elements common to the inefficient method (lid lifting and hole poking) to cumulatively produce the efficient solution demanded by the scenario used in Experiment 1? In Experiment 1 only two chimpanzees were observed to do this, with the majority instead sticking to their known behaviours despite potential gains in extraction efficiency. Now however, seven additional chimpanzees within the ‘social information’ groups and one from the ‘non-seeded’ groups had mastered use of an alternative, independent solution (door pull and extraction at point B), which could potentially be combined with other known behaviours (elements of the inefficient solution) to produce a compound technique that they were previously not seen to use when some of these elements were novel.

## Experiment 3: Modifying, inhibiting and building on existing behaviours

To investigate chimpanzees’ potential for such accumulation, the token was repositioned in the same location as in Experiment 1 (i.e. it was removed from the indent in the floor so its movement was no longer impeded), and could now be successfully extracted at either point A using the methods of E0, or from point B (Fig. 1). To extract from point B, individuals had to employ initial elements from their learned, inefficient technique (lid lifting and hole poking) but inhibit the remainder of the sequence resulting in extraction at point A and instead combine lid lifting and poking with the element unique to efficient extraction (the door pull at point B). Alternatively, individuals could now revert back to using their earlier well-practiced inefficient technique, with this method reliably yielding the token, but much more slowly.

## Experiment 3: Results

### Extractions within the test period

One individual in the ‘social information’ groups and three individuals in the ‘non-seeded’ groups chose not to participate during the test period (‘E3’– Supplementary Table S1).

### Switching behaviours

In the ‘social information’ groups, there was a significant change of behaviour from use of the earlier, trained inefficient method, with seven individuals now using the more efficient compound solution needed (One-tailed Wilcoxon signed rank test comparing percentage use of efficient behaviours: *Z* = −2.410, *n* = 10, P = 0.008, median_E0_ = 0%, median_E3_ = 88.2%, *r* = −0.54; Fig. 2). In the ‘non-seeded’ groups, one individual, *Kt,* also built on her prior solution to use the more efficient method. No additional individuals in the ‘non-seeded’ group used the efficient method of extraction, with four exclusively sticking with the inefficient solution.

**Figure 4:**
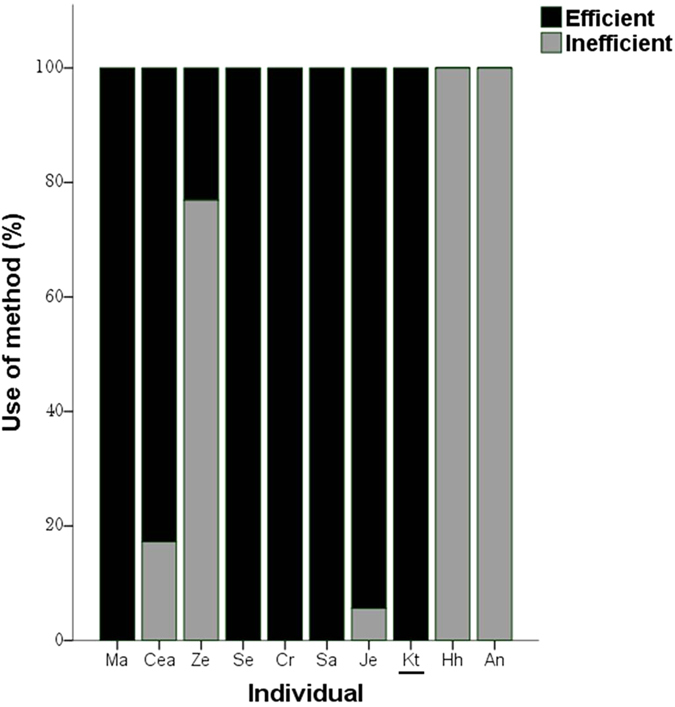
Percentage use of the inefficient and efficient solution of token extraction in Experiment 3 for each individual with prior experience of extraction via point B during Experiment 2. The ‘non-seeded’ individual *Kt* is underlined, with all other individuals being ‘social-information’ participants.

At the individual level, of those with personal experience of the efficient method (*n* = 9 ‘social information’ participants and *n* = 1 ‘non-seeded’ participant), seven showed a significant change of behaviour from their initial inefficient method to using the efficient compound solution (one-tailed Fisher exact tests with Bonferroni corrected P value = 0.005), whilst three reverted back to preferentially using the inefficient method (P > 0.005). In sum, five exclusively used the efficient method, three flexibly switched between using both methods, and two exclusively returned to the inefficient method (Fig. 4 and Table 2).

## Experiment 3: Discussion

Seven chimpanzees in the ‘social information’ groups now displayed the efficient solution employed by the models. Only two of these individuals had previously been seen to use this efficient solution, when this required the addition of a novel element, in E1. The other five, along with the innovator *Kt* in the non-seeded’ group, displayed a cumulatively built combination of elements they had learned in E0 and E2. From the results of E3 we conclude that accumulation involved the combination of behaviour routines already in the repertoire. One of these, opening the door at point B (even if it was the case that this was acquired only by affordance learning about the significance of this door, but also if it involved copying the action sequence involved), gave rise to behavioural routines that could be combined with parts of an earlier-acquired procedure, of opening lids and poking, learned via training in E0. Chimpanzees’ successes in E3 additionally displayed an ability to flexibly inhibit the remainder of the trained routine for extraction at point A. Such capacities for cumulative combination, although modest compared to full cumulative culture, could, we submit, provide important foundations for cumulative culture if present in ancestral states.

## General Discussion

Chimpanzees were trained to use a relatively laborious sequence of actions to extract a valuable food-token from a puzzle-box. This initial method was sufficiently complex to require socially-facilitated acquisition in most chimpanzees and we ensured it was then extensively practiced, to become routine, as in cultural behaviours in the wild. A different, more efficient alternative was then demonstrated by a high ranking female conspecific. This new solution involved partial use of behaviours in common with the established extraction technique as well as the addition of a novel element.

When chimpanzees could still successfully forage with their established method (in E1), only a small minority relinquished this and flexibly upgraded to the more efficient alternative witnessed. The predominant failure to switch to the more efficient technique is consistent with earlier reports of chimpanzee conservatism^13^,^16^,^18^,^19^,^20^,^21^ and may offer a partial explanation for the relative stasis of chimpanzee culture. However, when their established behaviours were made considerably more inefficient in E2, most chimpanzees observing a knowledgeable individual were able to relinquish their inefficient behaviour and flexibly switch to using an alternative strategy. When in E3 they were again challenged by the task configuration of E1, the majority of these chimpanzees showed an ability to build on prior behaviours by combining already acquired elements of their learned use of the door for extraction at point B and parts of their earlier technique for extraction at point A. They had not achieved this earlier in E1, when success required the addition of a *novel* behaviour to the sequence. The cumulative combinations recorded in E3 thus stand in contrast to the findings of previous studies where chimpanzees appear behaviourally inflexible^13^,^16^. Our results suggest that in certain contexts at least, chimpanzees may combine *known* behaviours to match an efficient compound technique demonstrated by others.

Although chimpanzees show a considerable degree of behavioural conservatism, we suggest these results indicate that they also have an ability to combine independent behaviours to produce more efficient compound action sequences. Such an ability, while not yet truly cumulative, may be one of the foundational abilities (or candidate mechanisms) for human cumulative culture, through the ability to “add an existing technique used in a different context ….to an existing technique, and integrate them functionally” (p.181^44^). This shares similarities with human studies in which recombination of behavioural variants is employed to move solutions closer to an optimum^45^,^46^,^47^,^48^,^49^,^50^; that is, accumulation may commonly be brought about through novel recombination of existing behaviours, creating “innovations without invention, creativity or trial and error learning” (p.5^49^).

Whilst we offer evidence for a potential core prerequisite of cumulative culture, this is not evidence of cumulative culture itself, as the behaviours of interest were also produced spontaneously by one chimpanzee we studied, and they do not require the combination of multi-generational contributions by several innovators, which is inherent to full-blown cumulative culture^10^. Further, our study was not designed to dissect exactly how the chimpanzees were learning from the available social information, whereas advanced cultural accumulation is thought to depend on high fidelity transmission^51^, as well as cognitively complex learning heuristics^15^,^52^. However, chimpanzees in our study were able to use multiple solutions as well as to build on and combine prior behaviours to efficiently solve an extractive foraging problem, indicating greater potential for cumulative change than found in many earlier studies and emphasized in recent reviews (e.g. ref. 53). The accumulation observed here lends support to the plausibility that some behaviour exhibited by wild chimpanzees is actually the result of a cumulative process, even if elementary compared to that observed in human culture^54^,^55^,^56^,^57^,^58^.

## Experiment 1: Methods

### Subjects and housing

N = 43 individuals (18 males; average age: 29.1; range: 11.9–50.5 years; Table 1) were group housed at the National Center for Chimpanzee Care at the Michale E. Keeling Center for Comparative Medicine and Research of The University of Texas MD Anderson Cancer Center in Bastrop, Texas, U.S.A. Group size ranged from 5–10 individuals. Chimpanzees were trained and tested in both their outside enclosures (ranging in size from corrals at 4,300 square feet to Primadomes^TM^ measuring approximately 34 feet in diameter and 25 feet high) and indoor dens (ranging in size from 6 feet deep by 15 feet wide to approximately 8 feet and 8 inches deep by 9 feet wide). Individuals were given the opportunity to voluntarily participate and separate from their group for further training and testing purposes in their inside enclosures for a period of no longer than 30 minutes. Participants were not food or water deprived during training or testing.

### Apparatus

A transparent, elongated, Plexiglas ‘Serialbox’, measuring 61 centimetres long, five centimetres high and five centimetres wide, was attached to a mobile cart and pushed to the mesh of enclosures. Along the length of the transparent Serialbox were four compartments (Fig. 1). Each compartment had a hinged lid on top which could be lifted open. Under each lid were four finger holes (2.5 cm in diameter) that permitted an object initially placed inside the box at the left-most end from the chimpanzees’ perspective to be pushed the length of the apparatus. This object could then be extracted through an opening at the other end of the Serialbox (‘Extraction point A’ in Fig. 1). A small door spanning two thirds of the first compartment was fitted on the chimpanzee side of the apparatus and could be pulled open using a handle protruding outside the box to give alternative and quicker access to the left-most compartment (‘Extraction point B’ in Fig. 1), where the token was initially positioned.

### Procedure

#### Training phase (5 groups, 38 chimpanzees)

Chimpanzees were initially trained to associate a small purple plastic token with a reward by trading this with experimenter SJD in exchange for one grape. The token was then placed inside the apparatus three quarters of the way along the first compartment (Fig. 1). The inefficient method of retrieving the token was demonstrated by SJD three times before participants interacted with the Serialbox. The inefficient method involved the lifting of each of the lids of the four compartments providing access to the finger holes. These holes were used to ferry the token along the compartments of the apparatus until it could be extracted from point A (Supplementary Video 1). Following these demonstrations, the box was pushed to the mesh allowing all individuals in each group access. Once the token was extracted from the apparatus, it was exchanged with SJD for one grape. During the training phase, the efficient method was not available because the pull door was locked shut, preventing extraction from point B. If an individual was not able to successfully retrieve the token after demonstrations, scaffolding of the solution was provided whereby the token was positioned adjacent to extraction point A until extraction from this point was mastered, with additional demonstrations given if necessary. The token was gradually placed further away until the chimpanzee was manoeuvring the token along the length of the apparatus by opening the lids and using the underlying finger holes. Participants were given the opportunity to engage with the Serialbox until all participating individuals had successfully retrieved the token a minimum of twenty times over no fewer than two training sessions. When an individual was successful in retrieving the token, the apparatus was pulled back from the mesh, reset and re-baited. If an individual showed interest in operating the apparatus but was unable to gain access due to monopolisation by more dominant individuals, they were offered the opportunity to voluntarily enter their indoor enclosures and participate by themselves until they had reached criterion for inclusion in the study.

#### Social information groups: Presence of social demonstrator (Three groups, N = 26)

##### Model training phase

After all participating chimpanzees had reached criterion, a high ranking female chimpanzee voluntarily separated from her group and was trained on how to solve the Serialbox using a more efficient method. This involved pulling the door open, and, due to the positioning of the token a short distance from the extraction point (Fig. 1), lifting one lid and using the underlying finger holes to manoeuvre the token towards point B for efficient retrieval (Supplementary Video 2). Training sessions lasted around twenty minutes.

##### Social demonstration phase

The Serialbox was re-introduced to the entire group with the efficient method no longer locked. The token could now be retrieved via either extraction point A or B. The model was called by name and vocally encouraged to demonstrate the efficient method, which all models complied with. Following each extraction, the token was exchanged with SJD for one grape. After each participant had witnessed at least ten demonstrations of the more efficient method over no fewer than two separate testing sessions, the entire group was given the opportunity to interact with the Serialbox. A demonstration was taken to occur if an individual was within two metres of the model and the potential observer’s head was orientated towards the apparatus. If a participating individual did not come into proximity with the model during the social demonstration phase, they were given the opportunity to voluntarily separate with the model and observe her actions. After the model had successfully retrieved the token, the apparatus was pulled away from the demonstrator, reset and re-baited.

##### Testing phase (N = 11)

The apparatus was presented over ten hours to all participating individuals with both the efficient and inefficient methods as viable strategies to extract the token. After each successful extraction, the apparatus was pulled away, reset and re-baited. To avoid cueing of responses, SJD occluded the apparatus and her hand movements with a sheet during interactions with the box. The apparatus was not made available to any non-participating chimpanzee (i.e. any individual who had not met criterion to be included in the study).

#### Non-seeded groups: No social demonstrator (Two groups, N = 12)

Control groups experienced the Training phase and Testing phase as above, but no model seeded knowledge of the more efficient method.

#### Naïve group (1 group, N = 5)

This control group was exposed to the apparatus with no prior knowledge of any solution over ten hours of open diffusion. Both the efficient and inefficient methods were viable extraction techniques.

## Experiment 2: Methods

Methods followed those outlined in the Testing phase of Experiment 1 Methods with the exception that the token was now placed in an indent in the floor located directly behind (from the chimpanzee’s perspective) extraction point B (Fig.1). This impeded movement of the token along the length of the apparatus. The ‘naïve’ group was not included in Experiment 2. Following Yamamoto *et al*.^26^, if individuals within the ‘social information’ groups failed to switch, they were provided with salient demonstrations of the efficient method by SJD after this second period of open diffusion (one individual did not receive human demonstrations as she did not wish to separate from her group). To avoid unnecessary voluntary separation of participants from their group, so long as a participant was able to gain access to the Serialbox, human demonstrations were given in the presence of other group members. If instead the participant struggled to gain access, they were offered the opportunity to voluntarily separate and given additional demonstrations over a period lasting no more than 30 minutes. After the participant attempted the inefficient method, SJD pulled the apparatus back and demonstrated use of the door. If participants were still attempting to use the inefficient method, SJD provisioned the apparatus with the door already open, facilitating extraction via point B.

## Experiment 3: Methods

The token was again placed inside the apparatus three quarters of the way along the first compartment (as in Experiment 1). The apparatus was presented over five hours to all participating chimpanzees (19 individuals across the ‘social information’ and ‘non-seeded’ control groups), with both the efficient and inefficient methods as viable strategies to extract the token, following the procedure outlined in the Testing phase of Experiment 1 Methods.

### Analyses

Records of the social demonstration and testing phases were both narrated and visually recorded using a HC-920 Panasonic camcorder. Responses were coded *in situ* for all groups, with ‘social information’ groups’ behaviour additionally coded through video analysis.

### Ethics Statement

Ethical approval was granted for this study by the UTMDACC Institutional Animal Care and Use Committee (IACUC approval number 0894-RN01) and the University of St Andrews’ Animal Welfare and Ethics Committee, and was carried out in accordance with approved guidelines.

## Additional Information

**How to cite this article**: Davis, S. J. *et al*. Foundations of cumulative culture in apes: improved foraging efficiency through relinquishing and combining witnessed behaviours in chimpanzees (*Pan troglodytes*). *Sci. Rep.* **6**, 35953; doi: 10.1038/srep35953 (2016).

## Supplementary Material

Supplementary Information

Supplementary Video 1

Supplementary Video 2

## Figures and Tables

**Table 1:** Demographics of participants meeting criterion for inclusion.

Individual	Sex	Age	Group	Wild/captive	Rearing
My	F	49.26	Model	Wild	Unknown
Ma	F	48.26	Social info	Wild	Unknown
Cea	F	23.10	Social info	Captive	Mother
Ze	F	11.95	Social info	Captive	Mother
Ta	F	21.36	Social info	Captive	Mother
Co	F	30.24	Model	Captive	Mother
Se	F	15.40	Social info	Captive	Mother
Hh	F	23.93	Social info	Captive	Mother
Cr	M	18.78	Social info	Captive	Mother
An	M	22.86	Social info	Captive	Mother
Mi	F	24.69	Model	Captive	Mother
Sa	F	24.71	Social info	Captive	Mother
Je	F	24.96	Social info	Captive	Mother
Si	M	24.74	Social info	Captive	Mother
Kt	F	25.78	Non-seed	Captive	Nursery
Na	F	23.88	Non-seed	Captive	Nursery
Ae	F	39.39	Non-seed	Wild	Unknown
Ai	F	19.21	Non-seed	Captive	Nursery
Gs	M	22.41	Non-seed	Captive	Nursery
Chu	F	33.57	Non-seed	Captive	Mother
Sha	F	23.75	Non-seed	Captive	Mother
Ka	F	23.56	Non-seed	Captive	Mother
Jy	M	42.52	Naïve	Wild	Unknown
Ua	F	50.53	Naïve	Wild	Unknown
Cy	M	24.41	Naïve	Captive	Mother
Zy	M	43.52	Naïve	Wild	Unknown
Ha	F	48.53	Naïve	Wild	Unknown

From left to right: Individual: Initials of participant (individuals are organised by their groups with participants listed under their respective models); Sex: F = female, M = male; Age: Age in years at time of testing; Group: Social info = social information group; Non-seed = non-seeded; Naïve = Naïve group; Captive/wild: Captive = born in captivity, Wild = born in the wild; Rearing: Mother = raised by mother, Nursery = raised by human caretakers.

**Table 2:** Summary of participant’s behaviour in the ‘social information’ groups as well as the innovator (*Kt*) in the ‘non-seeded’ group.

Individual	Exp 1	Exp 2	Human Demos	Exp 3
	Old solution somewhat inefficient	Old solution highly inefficient		Old solution somewhat inefficient
Sa	Build	Switch	N/A	Build
Se	Build/revert	Switch	N/A	Build
Ma	Stay	Switch	N/A	Build
Cea	Stay	Switch	N/A	Build
Ze	Stay	Switch	N/A	Build
Kt	Stay	Switch	N/A	Build
Cr	Stay	Stay	Switch	Build
Je	Stay	Stay	Switch	Build
Hh	Stay	Stay	Switch	Revert
An	Stay	Stay	Switch	Revert
Ta	Stay	Stay	N/A	Stay
Si	Stay	Stay	Stay	N/A

‘Build’ denotes building on the inefficient solution. ‘Stay’ denotes maintaining use of the inefficient solution. ‘Revert’ denotes reverting back to the inefficient solution after having efficiently extracted through point B. ‘Switch’ denotes relinquishing the inefficient solution in favour of using the door and extraction at point B. ‘N/A’ represents no participation in this phase.
