# Supplementary material for: Foundations of cumulative culture in apes: improved foraging efficiency through relinquishing and combining witnessed behaviours in chimpanzees (Pan troglodytes)
Source: Sci Rep. 2016 Oct 24;6:35953. doi: 10.1038/srep35953 (PMC5075880; doi:10.1038/srep35953)
Supplement: Supplementary Information [file srep35953-s1.pdf]

Foundations of cumulative culture in apes: improved foraging efficiency through relinquishing and combining witnessed behaviours in chimpanzees (*Pan troglodytes*)

Sarah J. Davis[1, 2], Gillian L. Vale[1, 2], Steven J. Schapiro[2], Susan P. Lambeth[2], Andrew Whiten[1, *]

[1]Centre for Social learning and Cognitive Evolution, School of Psychology & Neuroscience, University of St Andrews, St Andrews, Scotland

[2]National Center for Chimpanzee Care, Department of Veterinary Sciences, Michale E. Keeling Center for Comparative Medicine and Research, the University of Texas MD Anderson Cancer Center, Bastrop, TX 78602, USA

* Corresponding author's email: aw2@st-andrews.ac.uk

	Group	Total	Efficient	Median	Range
**Training**	Social	254	0	21	20-32
	Non-seeded	202	0	23	20-37
**Test period 1**	Social	496	189	22	2-179
	Non-seeded	299	0	39.5	4-81
**Test period 2**	Social	440	362	15	2-194
	Non-seeded	235	144	15	2-145
**Test period 3**	Social	418	324	13	0-117
	Non-seeded	154	84	9	0-84

Supplementary Table S1. Extractions by participants across test periods. ‘Total’ is the sum of extractions made by all participants in the ‘social information’ and ‘non-seeded’ groups; ‘Efficient’ is the sum of efficient extractions; ‘Median’ is the median number of extractions; ‘Range’ is the range of extractions.

	Model	Total	Median	Range	Efficiency
**Experiment 1**	My	90	24	13-29	33.3
	Co	65	18	11-18	4.1
	Mi	134	46	34-56	11.9
**Experiment 2**	My	97	24.5	11-37	70.1
	Co	8	1.5	1-4	90.2
	Mi	156	52	32-72	92.2

Supplementary Table S2. Summary of models' behaviours. Extraction data is presented based on group observations of their respective models. Models are identified by their initials. 'Total' is the sum of participants' observations of extractions made by their model. 'Median' is the median number of observed extractions made by the model. 'Range' is the range of the sum of participant observations of extractions made by the model. For Experiment 1, the total, median and range figures include extractions observed during the social demonstration phase in addition to those observed during Experiment 1 testing period. 'Efficiency' (in seconds) is the difference of the model's mean latency to extraction relative to the median of participants' mean investment in using the inefficient method (i.e. includes times spent performing the inefficient method whether the participant was successful or not in extracting the token), and reflects how much faster the efficient method was compared to the inefficient.

Individual	Group	Exp1: Obs	Exp1: Extract	Exp2: Obs	Exp2: Extract	HD: Obs	HD: Extract	Switch: Obs	Switch: Extract
**Sa**	Social info	**21**	**0**	N/A	N/A	N/A	N/A	21	1
**Se**	Social info	**15**	**6**	N/A	N/A	N/A	N/A	15	7
**Ze**	Social info	29	4	**15**	**4**	N/A	N/A	44	9
**Cr**	Social info	11	10	2	3	**17**	**16**	30	30
**Ma**	Social info	27	33	**15**	**2**	N/A	N/A	42	36
**An**	Social info	19	16	5	2	**17**	**16**	41	35
**Je**	Social info	57	18	102	14	**10**	**9**	169	42
**Kt**	Non-seed	0	81	**0**	**1**	N/A	N/A	0	83
**Cea**	Social info	13	67	**9**	**17**	N/A	N/A	22	85
**Hh**	Social info	18	119	1	19	**12**	**11**	31	150
**Si**	Social info	45	2	49	7	12	12	106	No switch
**Ta**	Social info	21	22	18	3	N/A	N/A	39	No switch
**Na**	Non-seed	0	49	3	20	N/A	N/A	3	No switch
**Ae**	Non-seed	0	4	2	7	N/A	N/A	2	No switch
**Ai**	Non-seed	0	55	0	20	N/A	N/A	0	No switch
**Gs**	Non-seed	0	58	0	2	N/A	N/A	0	No switch
**Chu**	Non-seed	0	30	0	18	N/A	N/A	0	No switch
**Sha**	Non-seed	0	6	0	12	N/A	N/A	0	No switch
**Ka**	Non-seed	0	16	0	11	N/A	N/A	0	No switch

Supplementary Table S3: Observation and extraction data for each participant. From left to right: 'Individual' is the participant represented by their initials and organised in ascending order of total number of inefficient extractions experienced before switching to the efficient solution (final column); Group: 'Social info' = 'social information' group; 'Non-seed' = 'non-seeded' group. 'Exp1: Obs' and 'Exp2: Obs' are the number of observations of the efficient method for each individual before they switched to the efficient method in each experimental condition respectively. 'Exp1: Obs' includes observations from the 'social demonstration phase'. 'Exp1: Extract' and 'Exp2: Extract' are the number of extractions in which a participant successfully extracted or attempted to extract the token before switching to use of the efficient method. 'N/A' denotes the data during this experimental phase are not applicable to the participant's switching behaviour. 'HD: Obs' and 'HD: Extract' are the number of observations and extractions experienced in the 'Human Demonstration' phase of Experiment 2 respectively. 'Switch: Obs' is the total number of observations across experimental phases before switching to the efficient method. These data include observations of the model, of other participants and, where applicable, human demonstrations of the efficient method. If the individual did not switch, this number is the total number of observations across experimental phases. 'Switch: Extract' refers to the extraction attempt the individual switched on and includes extractions across all experimental phases. The experimental phase in which the participant switched is reflected with the emboldening and underlining of the number of efficient extractions observed and the number of extractions experienced pre-switch.

Supplementary Video 1. Inefficient method. A chimpanzee demonstrates use of the inefficient extraction method.

Supplementary Video 2. Efficient method. A chimpanzee demonstrates use of the efficient extraction method.
